# The caries impacts and experiences questionnaire for Turkish children by age groups’

**DOI:** 10.1186/s12903-023-03321-3

**Published:** 2023-08-24

**Authors:** Sacide Duman, Feyza Inceoglu

**Affiliations:** 1 Department of Paediatric Dentistry, Faculty of Dentistry, Inonu University, Malatya, 44280 Turkey; 2 Department of Biostatistics, Faculty of Medicine, Malatya Turgut Ozal University, Malatya, Turkey

**Keywords:** Caries Impacts and Experiences Questionnaire, Dental caries, Quality of life

## Abstract

**Background:**

The Caries Impacts and Experiences Questionnaire for Children (CARIES-QC) assess children’s effects of dental caries on their quality of life. This study aimed to determine the scale’s Turkish version (CARIES-QC/T) validity and reliability according to age groups and to create the scale that is specific to selected age groups.

**Methods:**

Children were divided up into 3 age groups (5–7, 8–10 and 11–14 ages). Explanatory factor analysis (EFA) was used in the concept validation process. Confirmatory factor analysis (CFA) was used for cross-cultural validity. For each group, test-retest analyses were performed on 20 children. Inter-question correlation and Cronbach’s alpha were used to examine internal consistency.

**Results:**

A total of 360 children (mean age; 9.04, 56.1% girls,) 120 children in each group, participated in the study. Questions 7 and 12 for the 5–7 age group, Questions 4 and 7 for the 8–10 and 11–14 age groups were excluded from the analysis (according to EFA results; factor loads < 0.30). Three modified CARIES-QC/T scales structures with a total of 10 questions were developed for the age groups of 5–7, 8–10, and 11–14.

**Conclusions:**

Some questions on the Caries-QC/T scale should be eliminated, it was found when it was evaluated for age specificity. Although the results of the Caries-QC scale studies to be carried out in different societies and age groups vary, the high sample size in this study and the statistically strong results showed that the Caries-QC/T scale forms that we adapted could be used by the specified age groups.

## Background

The term health-related quality of life refers to the capabilities of individuals to perform their life functions and the ways in which they perceive the physical, social and psychological spaces in their lives [1]. Oral health-related quality of life (OHRQoL), which is gaining popularity in paediatric dentistry, describes a concept that aims to evaluate the functional and psychosocial outcomes of oral health [2]. The presence of oral pathologies such as caries [3], trauma [3, 4], and malocclusion [3] among developing children, as well as their previous experiences and fears [5] with regard to dentistry, can have negative effects and reduce the quality of their lives. Previous studies that examined OHRQoL in children and young adults [3, 6] have reported that dental caries resulted in negative consequences related to conditions such as acute infections, nutritional deficiencies, pain while brushing, and sleep disturbances.

Because children are often unable to evaluate their emotions, scales have been developed to help parents and caregivers assess the children’s perceptions with regard to OHRQoL [7]. However, recent studies [8–10] that were conducted on children have indicated that they are, in fact, able to express their emotions and that it is important to evaluate these. Although various scales have been developed for children to evaluate their OHRQoLs, these scales [8, 9, 11] have been designed to determine the effects that are associated with different orofacial conditions and may, therefore, not be sensitive enough to assess the effects that are related specifically to dental caries.

The Caries Impacts and Experiences Questionnaire for Children (CARIES-QC) is a child-centred, caries-specific OHRQoL scale, with 12 questions and one global question, that Gilchrist et al. [12] developed to evaluate how children perceive the impacts of caries and the effects that caries have on their qualities of life. Since the validity and reliability of this scale was established, it has been translated into Chinese [13], Dutch [14], Arabic [15], and Turkish [16], and is used in many different countries. The scale was designed so that all children from 5 to 16 years of age could understand it. However, taking into account the developmental differences among children in terms of their roles and cognitive abilities, the validity and reliability of the Turkish version of the CARIES-QC (CARIES-QC/T), according to age groups have not been determined. This study aimed to determine the CARIES-QC/T scale’s validity and reliability according to age groups and to create the scale that is specific to selected age groups.

## Methods

### Ethical considerations

Approval for the study was obtained from the Inonu University Ethical Committee of Non-Invasive Research (Decision No: 2022/3018).

### Participants

The study included children who had applied to the Department of Paediatric Dentistry from January to June 2022. The children’s parents signed informed consent forms after being told about the study. Children of parents who did not sign informed consent forms and whose parents did not consent to participate in the study were excluded from the study. The inclusion criteria were children aged 5 to 14 years who had active dental caries. Children with oral diseases (conditions) other than dental caries, such as dental trauma, cleft lip and palate and craniofacial abnormalities, and children who could not understand the scale questions even with support were not included in the study. First, the demographic data, including age, gender, education levels and places of residence, were documented. The CARIES-QC that Gilchrist et al. [12] developed was used to determine the OHRQoLs of the children. The study used the Turkish version of the CARIES-QC (CARIES-QC/T), which was adapted into Turkish by Uslu and Bani [16] and tested for validity and reliability. After the literature review [8, 9, 12], the children were divided into three age groups, according to their developmental stages: 5 to 7 years, 8 to 10 years and 11 to 14 years. The children who were aged 8 to 10 and 11 to 14 completed the CARIES-QC/T themselves in the waiting room. The CARIES-QC/T was read to the children who were 5 to 7 years of age, and their responses were filled in by the researcher. When a participant had a question, the researcher was consulted, and the researcher answered the question in simple terms so that this did not affect the child’s response. Each assessment took about 10 min to complete.

Once the participants had finished the CARIES-QC/T, their teeth were dried with compressed air and then examined under a reflector lamp with a mouth mirror and a dental probe. All examinations were done in the dental office. For standardization purposes, all examinations were conducted by a single experienced dentist (S.D.). Assessment of caries in primary and permanent teeth was made using the decayed, missing and filled teeth (dmft and DMFT) index, which is recommended by the WHO. Missing teeth that had been lost for reasons other than caries, such as because of trauma or physiological tooth extraction, were not included in the DMFT scores.

### Measures

The CARIES-QC/T consists of 12 questions in addition to one that was introduced to examine convergent validity, “How much of a problem are your teeth for you?“. The answers to all questions were planned to include, ‘Not at all’, ‘A bit’ and ‘A lot’, with respective scores ranging from 0 to 2. A three-point Likert scale was implemented. Higher scores indicate a higher effect, ranging from 0 to 24 points.

### Statistical analysis

The sample size was calculated according to the method recommended by Terwee et al. [17] The sample number for factor analysis should be seven times the number of questions (12 questions × 7 = 84), with at least 100 participants. The Mahalanobis distance method included in the AMOS package program was used for multivariate normal distribution analysis, a basic assumption of multivariate analysis methods. Exploratory factor analysis (EFA) was used to analyse the dataset that had been prepared for the study during the concept validation phase. Before the factor load distribution and percentages of explained variance through EFA were calculated, Bartlett’s test of sphericity was conducted to determine the sample structure’s suitability to the scale. A Kaiser-Meyer-Olkin (KMO) test was used to determine whether the sample size was sufficient for EFA. Next, confirmatory factor analysis (CFA) was used to assess the model’s cross-cultural validity established through EFA. The chi-square-to-degrees-of-freedom ratio (χ^2^/sd), root mean square error of approximation (RMSEA) and goodness of fit index (GFI), normed fit index (NFI), Incremental Fit Index (IFI) and comperative fit index (CFI) goodness-of-fit indexes were used to assess the CFA model’s suitability. Reliability analyses and test-retest analyses were then conducted. Internal consistency was assessed using Cronbach’s α and inter-question correlation. EFA, reliability analysis and test-retest analyses were performed using Statistical Package for the Social Sciences (SPSS) Version 26. Meanwhile, the Amos 24 package program was used for CFA. For the model’s final version, test and goodness-of-fit values were calculated. A significance level (α) of *p* = 0.05 was adopted in the applied analyses.

## Results

### Participant characteristics

In total, 360 children completed Caries-QC/T, 56.1% of whom were girls and 43.9% of whom were boys and the sample size was found to be sufficient. On average, respondents were 9.04 ± 2.48 years old. Their mean dmft and DMFT indexes were 2.27 ± 2.59 and 5.41 ± 4.55, respectively. Respondents’ demographic, dmft and DMFT data are presented in Table 1.

**Table 1:** Demographic information of the participants and DMFT, dmft scores

Groups	Total	5–7 Age	8–10 Age	11–14 Age
	n (%)	n (%)	n (%)	n (%)
**Girl**	202 (56.1)	72 (60.0)	65 (54.2)	65 (54.2)
**Boy**	158 (43.9)	48 (40.0)	55 (45.8)	55 (45.8)
**Total**	**360 (100.0)**	**120 (33.3)**	**120 (33.3)**	**120 (33.3)**
	**Mean ± sd**	**Mean ± sd**	**Mean ± sd**	**Mean ± sd**
**Age**	9.04 ± 2.48	6.26 ± 0.74	8.85 ± 0.76	12.01 ± 12
**dmft**	5.41 ± 4.55	9.1 ± 4.00	6.22 ± 2.84	0.9 ± 0.00
**DMFT**	2.27 ± 2.59	0.53 ± 1.61	2.03 ± 1.76	4.24 ± 4

n; frequency, %; percent, sd; standart deviation

### Validity assessment

The multivariate normal distribution index was calculated with the Amos program according to the Mahalanobis distance method. Since this index was calculated as less than 8 for the respondent groups (5-to-7-year-old group: 2.957; 8-to-10-year-old group: 2.541; 11-to-14-year-old group: 4.823), multivariate normal distribution was assumed [18].

An increase in the Bartlett’s sphericity test value indicated an increase in the data’s suitability for EFA. The lowest KMO value required to apply EFA is 0.60, and a value of 0.81–0.90 is defined as ‘very good’ [19]. Therefore, the sample structure and constituent scale models were found to be suitable for EFA (Table 2).

**Table 2:** Bartlett Test of Sphericity and Kaiser-Meyer-Olkin (KMO) test for Explanatory Factor Analysis (EFA).

		5–7 Age	8–10 Age	11–14 Age
**Kaiser-Meyer-Olkin (KMO)**		0.843	0.835	0.854
**Barlett’s Test of Sphericity**	**Test**	349.954	481.406	542.208
	**df**	78	78	78
	**p**	< 0.001*	< 0.001*	< 0.001*

df; degrees of freedom

The factor loadings, mean and standard deviation values of the questions obtained from the EFA results, which were applied to the data sets, are shown in Table 3.

**Table 3:** Descriptive statistics and item factor loads

Items	5–7 Age		8–10 Age		11–14 Age	
	Mean ± sd	Factor Loads	Mean ± sd	Factor Loads	Mean ± sd	Factor Loads
**1.Hurts**	1.02 ± 0.69	0.731	0.97 ± 0.65	0.690	0.78 ± 0.69	0.785
**2.Hard to eat some foods**	0.74 ± 0.72	0.611	0.79 ± 0.68	0.576	0.68 ± 0.63	0.607
**3.Eating on one side**	1.08 ± 0.84	0.547	1.03 ± 0.8	0.676	0.89 ± 0.75	0.681
**4.Food stuck**	1.18 ± 0.65	0.427	1.18 ± 0.66	***0.290***	1.01 ± 0.64	***0.283***
**5.Kept awake**	0.33 ± 0.58	0.498	0.42 ± 0.69	0.597	0.3 ± 0.6	0.773
**6.Annoyed**	0.65 ± 0.69	0.635	0.78 ± 0.7	0.719	0.64 ± 0.66	0.762
**7.Hurt when brushing teeth**	0.37 ± 0.56	***0.291***	0.4 ± 0.61	***0.278***	0.49 ± 0.62	***0.280***
**8.Eating carefully**	0.85 ± 0.77	0.642	0.89 ± 0.83	0.702	0.74 ± 0.73	0.732
**9.Eating slowly**	0.85 ± 0.78	0.672	0.68 ± 0.76	0.648	0.47 ± 0.66	0.721
**10.Feeling cross**	0.57 ± 0.73	0.600	0.49 ± 0.69	0.588	0.57 ± 0.72	0.418
**11. Cried**	0.58 ± 0.74	0.600	0.7 ± 0.78	0.688	0.47 ± 0.67	0.635
**12. Hard to do schoolwork**	0.17 ± 0.42	***0.298***	0.27 ± 0.5	0.514	0.22 ± 0.49	0.526
**% Total Explained Variance**	**Total = 51.553**		**Total = 55.055**		**Total = 57.615**	

sd; standard deviation

The lowest possible factor load value calculated for the scales was 0.30 [20]. Because their factor loads were less than 0.30, the following questions were excluded from further analysis: Question 7 and Question 12 for the 5-to-7-year-old respondent group, Question 4 and Question 7 for the 8-to-10-year-old respondent group and Question 4 and Question 7 for the 11-to-14-year-old respondent group. Three short version structures with a total of 10 items were developed for the age groups of 5–7 (Caries-QC/T _5-7_), 8–10(Caries-QC/T _8-10_), and 11–14 (Caries-QC/T _11-14_).

**Fig. 1:**
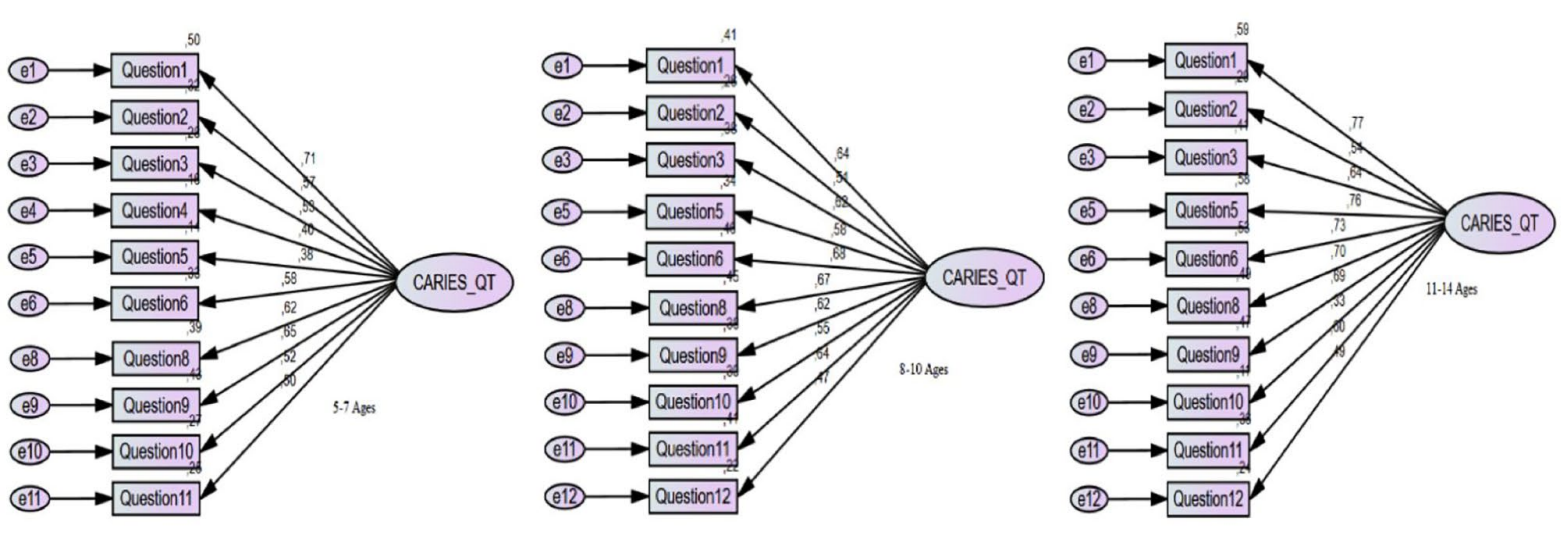
Confirmatory factor analysis (CFA) diagram of the short versions Caries-QC/T scale divided into age groups

CFA was applied to all three respondent groups’ samples to test whether the scale was correct for the model calculated by EFA and to validate the established models’ scale structures. A model diagram of the scales is presented in Fig. 1.

Because the models’ χ^2/^sd ratio was less than 3, the model was found to be perfectly statistically compatible. Additionally, because the GFI, NFI, IFI and CFI goodness-of-fit index values exceeded 0.90, the model was found to be compatible. An RMSEA value of less than 0.080 showed that the number of samples was sufficient for the model [21, 22]. The values acquired via the CFA model for the three sample groups are presented in Table 4.

**Table 4:** Goodness of Fit Coefficients and Accepted Value Ranges Calculated by the Confirmatory Factor Analysis (CFA) of the Caries-QC/T Scale

Index	5–7 Age		8–10 Age		11–14 Age		Acceptable Fit
	Model 1	Model 2	Model 1	Model 2	Model 1	Model 2	
**CMIN**	36.106	23.335	83.261	35.851	79.254	39.477	It is more compatible with the model with the smaller value.
**χ** ^**2**^ **/ df**	1.032	0.700	2.739	1.156	2.264	1.234	3–5
**IFI**	0.995	0.996	0.866*	0.987	0.900	0.983	0.90–0.95
**NFI**	0.871*	0.917	0.789*	0.909	0.835*	0.918	0.90–0.95
**CFI**	0.995	0.996	0.862*	0.986	0.898*	0.984	0.90–0.95
**GFI**	0.940	0.962	0.862*	0.944	0.888*	0.940	0.90–0.95
**RMSEA**	0.016	0.003	0.080*	0.036	0.103*	0.044	0.05–0.08

* The obtained values are insufficient for model fit

An examination of the CFA model’s goodness-of-fit indexes revealed that the NFI value for Caries-QC/T _5−7_, the GFI, CFI, NFI, IFI and RMSEA values for Caries-QC/T _8−10_ and the GFI, CFI, NFI and RMSEA values for the Caries-QC/T _11−14_ did not lie within the preferred range. If any goodness-of-fit index values obtained through analysis fall short of or exceed the accepted values, constructing a new model becomes difficult [23]. The established model can then be adjusted and improved using the modification indexes obtained from applied analyses. Modifications are considered necessary when multiple structures are used, when errors occur without measuring the structures’ correlations with indicators and when established relationships between residual terms that correlate with structures cannot be analysed [24].

In the current study, the model was modified by determining covariances between existing binary residual terms in order to reduce the RMSEA value to the desired level. The covariances used for this modification represented an concept that was not explained by the residual terms associated with two questions in the scale [25].

**Fig. 2:**
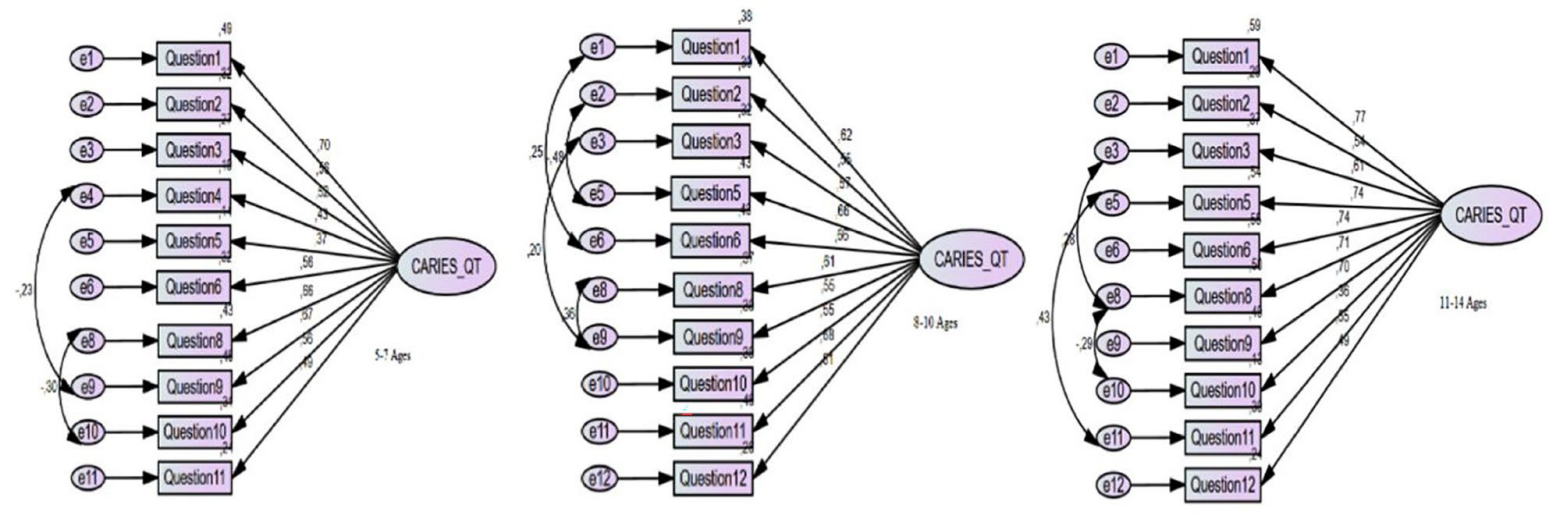
Path Diagram of short versions Caries-QC/T Scale with modification

While the coefficient controls of the model’s binary residual terms were modified, for Caries-QC/T _5−7_, the two residual terms with the highest correlation were Question 4–Question 9 and Question 8–Question 10. Meanwhile, for Caries-QC/T _8−10_, the residual terms with the highest correlation were Question 1–Question 6, Question 2–Question 5, Question 3–Question 9 and Question 8–Question 9. Finally, for Caries-QC/T _11−14_, the residual terms with the highest correlation were Question 3– Question 8, Question 5– Question 11 and Question 8–Question 10. Model diagrams established using the covariance between binary residual terms for all three models are presented in Fig. 2. The increase in the index values’ goodness-of-fit acquired from the models demonstrated that the modified model had an adequate scale structure [21, 22].

### Reliability Assessment

A Cronbach’s α coefficient and inter-question correlation coefficients are used to perform internal consistency analysis for scales with few questions. A value close to 0 indicates low reliability, while a value closer to 1 indicates high reliability. The closer a value is to 1, the more reliable it is. The lowest acceptable value for the correlation coefficient between a scale’s questions is 0.20 [26].

The Cronbach’s α coefficients calculated for the modified Caries-QC/T scales’ three respondent groups, as well as the correlation coefficients between the questions, are depicted in Table 5. The modified scale’s calculated value was between 0.81 and 0.90, which revealed that the scale had good reliability.

**Table 5:** Cronbach α coefficient and inter-item correlation coefficients

Items	5–7 Age		8–10 Age		11–14 Age	
	Corrected Item- Total Correlations	Cronbach α	Corrected Item- Total Correlations	Cronbach α	Corrected Item- Total Correlations	Cronbach α
**1.Hurts**	0.635	0.810	0.587	0.837	0.699	0.845
**2.Hard to eat some foods**	0.509		0.480		0.533	
**3.Eating on one side**	0.459		0.597		0.586	
**4.Food stuck**	0.325					
**5.Kept awake**	0.380		0.503		0.690	
**6.Annoyed**	0.524		0.610		0.671	
**7.Hurt when brushing teeth**						
**8.Eating carefully**	0.559		0.597		0.637	
**9.Eating slowly**	0.560		0.580		0.641	
**10.Feeling cross**	0.465		0.471		0.320	
**11. Cried**	0.475		0.585		0.512	
**12. Hard to do schoolwork**			0.427		0.412	

Test-retest reliability analyses were conducted to test whether the modified Caries-QC/T scales’ characteristics changed over time. Twenty samples were used for test-retest reliability analysis. The results of these tests are presented in Table 6. These analyses and applications showed that the developed Caries-QC/T _5−7_, Caries-QC/T _8−10_, and Caries-QC/T _11−14_ scales were valid and reliable for the three age groups.

**Table 6:** Test-Retest Analysis of the CARIES-QC/T.

		Mean ± sd	Cronbach α	t	p^1^	r	*p* ^*2*^
**5–7 age**	**Test**	8.15 ± 4.48	0.817	-0.123	0.904	0.914	< 0.001*
	**Re-Test**	8.2 ± 3.91	0.778				
**8–10 age**	**Test**	5.7 ± 2.96	0.711	-1.339	0.196	0.846	< 0.001*
	**Re-Test**	6.2 ± 3.05	0.718				
**11–14 age**	**Test**	4.85 ± 4.11	0.853	0.001	1.000	0.911	< 0.001*
	**Re-Test**	4.85 ± 4.38	0.891				

sd; standard deviation, *p < 0.05; r; pearson correlation coefficient, t; two paired samples t test

### Scale scoring

Modified Caries-QC/T scale is a Likert-type scale. Since the scale includes 10 questions for each respondent group with a 0–2-point scoring system, the lowest score that can be obtained is 0, while the highest possible score is 20. The average scores obtained for Caries-QC/T _5−7_, Caries-QC/T _8−10_ and Caries-QC/T _11−14_ groups were 7.83 ± 4.39, 7.01 ± 4.63 and 5.76 ± 4.45, respectively.

## Discussion

Despite dental caries’ widespread prevalence, few efforts have been taken to find out how this disease affects children’s daily life from the children themselves. Determining the prevalence of pain in child populations has been the main focus of investigations on the effects of dental caries. Although pain is unquestionably a significant consequence of caries, attention should also be paid to the wider psychosocial elements of this widespread condition [12].

The effect of dental health on quality of life can be measured using a variety of metrics [8, 9, 11, 27, 28]. Existing self-report OHRQoL surveys include the ‘generic’ measurement flaw, which means they are made to quantify the effects of all oral disorders on children’s life. In randomised controlled trials with a genuine underlying therapeutic impact, Wiebe and colleagues discovered that disease-specific instruments were more responsive to changes in health-related quality of life than were generic instruments [29]. Therefore, disease-specific measures are better at measuring changes in people with a particular disease, even while generic measures are beneficial for comparing populations and can be used to compare groups with diverse health problems. Caries-QC is a simple, short quality-of-life scale that covers a wide range of respondent ages, caries-specific measures and is based on child respondents’ answers [12]. Foster Page et al. compared Child Perception Questionnaire 11–14 (CPQ11–14), The Child Oral Health Impact Profile (COHIP) and Caries-QC to evaluate the effect of children’s caries condition on OHRQoL, determining that all three scales had acceptable internal reliability and middle-levelled, positive correlations between their scores [27]. A randomised, controlled study by Arrow et al. used The Early Childhood Oral Health Impact Scale (ECOHIS) and Caries-QC with Australian Aboriginal children, and the authors noted that both scales were found to be acceptable, reliable and valid [28]. Gilchrist et al. developed the Caries-QC scale in a study and determined that, compared to CPQ11–14, Caries-QC more strongly correlated with clinical data. These authors also found that Caries-QC could more sensitively evaluate effects related to dental caries [12].

Caries-QC scale was developed as a single 12-item scale for children aged 5 to 16 years. To make the scale understandable for all children in this age range, its design targeted the youngest children [12]. However, as a result of their ongoing cognitive, emotional, social, and linguistic development, children’s self-concept and health cognitions are age dependent. Similar to how daily activities change as children become older, so do their impressions of relationships, their understanding of emotional states, and their communication skills [8, 9]. According to Jokovic et al.‘s studys’, given these developmental variations, it cannot be develop a single, standardized self-report health status measure for children between the ages of 6 and 14. Instead, age-specific questionnaires for children ages 6–7, 8–10, and 11–14 are necessary. Because it is believed that these groups’ duties and cognitive abilities are similar [8]. Actually, the Caries-QC original scale has been translated into a variety of languages, and it has been assessed that these translations are applicable [13–16].but scale studies to be carried out in various societies and age groups may produce varying results. Nonetheless, previous studies about Caries-QC [12–16] have calculated the scale’s validity and reliability for all age groups in general without dividing respondents into separate age groups. Uslu and Bani [16] created the Caries-QC/T version in their study, and their validity and reliability analysis revealed that the scale’s Turkish version was applicable, but there was no evaluation by dividing it into age groups. However, the average age of starting school in our country is ≥ 5.5. It was believed that certain children with caries between the ages of 5 and 5.5 who did not attend school would not be able to provide a trustworthy response to the statement “hard to do schoolwork’ " (question 12) in the Caries-QC/T scale. The purpose of the study was to assess the intelligibility of the scale when viewed in terms of age groups because deleting a question from the scale or responding it wrongly will significantly influence its validity and reliability. According to this study results; modified scales had to be developed in order to apply the CARIES-QC/T scale to Turkish children in the specified age groups. From this point of view, our study contains important results.

To make the original scale understandable for all children in the targeted age range, its design incorporated the words these children use most to describe caries-related symptoms and experiences [12]. Roger et al. planned an anecdotal study aimed to use a classification system for paediatric-situation-specific, preference-based measurements based on Caries-QC and to validate this system with children. In that study, children expressed some uncertainty as to whether the ‘food stuck’ question were related to food getting stuck in their teeth in general or food getting stuck in the gaps between their teeth. The ‘food stuck’ question introduced translatability concerns when it was translated into other languages. Moreover, anecdotal evidence indicated that children may differently understand the ‘hard to do schoolwork’ question. Therefore, the authors thought these two questions could be excluded from their classification [30]. Furthermore, they thought that the scale could cover a ‘hard to do schoolwork’ response due to a toothache elsewhere in the ‘hurt’ category. Additionally, parent representatives thought that the pain related to ‘hurt when brushing teeth’ could also be evaluated under the ‘hurt’ question (Question 1) [30]. As a result of the EFA analysis applied in the current study, ‘hurt when brushing teeth’ and ‘hard to do schoolwork’ for group Caries-QC/T _5−7_ group, ‘food stuck’ and ‘hurt when brushing teeth’ for group Caries-QC/T _8−10_ and Caries-QC/T _11−14_ were excluded because their factor loads were < 0.30 [20] and this supports Roger’s anecdotal study [30]. Additionally, some children with caries between the ages of 5 and 5.5 who did not go to school were not included in the study because they could not answer the question ‘hard to do schoolwork’ in the Caries-QC/T scale. This not only reduced the size of our sample, but also limited our assessment of 5-5.5-year-olds who were out of school. In this study, the Caries-QC/T 5–7 scale, which was prepared by removing the “hard to do schoolwork” question; it allowed all children between the ages of 5–7 to be evaluated.

CFA is a structural equation modeling analysis method, but it is considered insufficient as it only provides a goodness of fit index value to test the accuracy of the models in analyses using structural equation modeling analysis [23]. Therefore, multiple goodness-of-fit index values are simultaneously used in the model structures with which accuracy is tested. To thus evaluate a model, evaluations should consider multiple values simultaneously instead of single values. All obtained index coefficients must reach the desired level (≥ 0.90) [23]. However, in the current study, an examination of the CFA model’s goodness-of-fit indexes revealed that the NFI value for the Caries-QC/T _5−7_ group, the GFI, CFI, NFI, IFI and RMSEA values for the Caries-QC/T _8−10_ group and the GFI, CFI, NFI and RMSEA values for the Caries-QC/T _11−14_ group did not within the preferred range (≥ 0.90). Therefore, the model was modified and determined to fit well through goodness-of-fit index values ≥ 0.90, which were calculated to evaluate the model’s significance in structural equation modelling analyses.

The Cronbach’s α coefficient and inter-question correlation coefficients are used to perform internal consistency analysis for scales with few questions. The Cronbach’s α values for the Caries-QC/T _5−7_, Caries-QC/T _8−10_ and Caries-QC/T _11−14_ groups were 0.810, 0.837 and 0.845, respectively, displaying very good internal consistency [26]. However, these values were low compared to previous studies’ corresponding values [12–16].

Discussions concerning the number of possible responses that should be used in Caries-QC have suggested that more options may increase sensitivity, but fewer options may increase reliability [31]. The authors believe that a three-point scale can lessen the load of the scale on the participants because the Caries-QC is intended for use with children of various literacy levels and across a wide range of ages.

The current study faced some limitations. Respondents in Caries-QC/T’s three age groups had presented at our clinic with complaints of active caries; hence, our study was not randomised. Similarly, we were unable to examine Caries-QC/T’s responsiveness since this assessment required a longitudinal study. Additionally, because our clinic only accepts patients aged 14 years or younger, we were unable to assess respondents in the 14-to-16-year-old group.

## Conclusions

Some questions on the Caries-QC/T scale should be eliminated, it was found when it was evaluated for age specificity. Although the results of the Caries-QC scale studies to be carried out in different societies and age groups vary, the high sample size in this study and the statistically strong results showed that the Caries-QC/T scale forms that we adapted could be used by the specified age groups.

## Data Availability

The datasets used and/or analysed during the current study available from the corresponding author on reasonable request.
